# An Indoor Location-Based Augmented Reality Framework

**DOI:** 10.3390/s23031370

**Published:** 2023-01-26

**Authors:** Jehn-Ruey Jiang, Hanas Subakti

**Affiliations:** Department of Computer Science and Information Engineering, National Central University, Taoyuan City 320317, Taiwan

**Keywords:** augmented reality, indoor localization, context-awareness

## Abstract

This paper proposes an indoor location-based augmented reality framework (ILARF) for the development of indoor augmented-reality (AR) systems. ILARF integrates an indoor localization unit (ILU), a secure context-aware message exchange unit (SCAMEU), and an AR visualization and interaction unit (ARVIU). The ILU runs on a mobile device such as a smartphone and utilizes visible markers (e.g., images and text), invisible markers (e.g., Wi-Fi, Bluetooth Low Energy, and NFC signals), and device sensors (e.g., accelerometers, gyroscopes, and magnetometers) to determine the device location and direction. The SCAMEU utilizes a message queuing telemetry transport (MQTT) server to exchange ambient sensor data (e.g., temperature, light, and humidity readings) and user data (e.g., user location and user speed) for context-awareness. The unit also employs a web server to manage user profiles and settings. The ARVIU uses AR creation tools to handle user interaction and display context-aware information in appropriate areas of the device’s screen. One prototype AR app for use in gyms, Gym Augmented Reality (GAR), was developed based on ILARF. Users can register their profiles and configure settings when using GAR to visit a gym. Then, GAR can help users locate appropriate gym equipment based on their workout programs or favorite exercise specified in their profiles. GAR provides instructions on how to properly use the gym equipment and also makes it possible for gym users to socialize with each other, which may motivate them to go to the gym regularly. GAR is compared with other related AR systems. The comparison shows that GAR is superior to others by virtue of its use of ILARF; specifically, it provides more information, such as user location and direction, and has more desirable properties, such as secure communication and a 3D graphical user interface.

## 1. Introduction

An indoor location-based augmented reality framework (ILARF) that combines indoor localization (IL) [1] and augmented reality (AR) [2] is proposed to facilitate the development of indoor AR systems for mobile devices, such as smartphones and tablets. IL is a technique to locate a device or user in an indoor environment. The location information provided by IL is indispensable in many applications, e.g., indoor navigation, tracking, and path planning [3,4,5]. AR is a technique to offer extra information about physical environments. It can provide users with an expansion of their unaided perception of the physical world. Many studies have applied AR in different areas such as training, digital learning, industrial design, and entertainment [6,7,8].

Several IL-AR studies [9,10,11,12,13,14] were conducted using different methods. Baek et al. [9] proposed AR for facility management with image-based IL computation. This system uses a Microsoft Hololens to perform AR and a high-end server to perform the deep-learning computations for image-based IL. Mobile IL-AR that uses a pyramidal beacon landmark was proposed by An et al. [10]. The system uses YOLO v3 to perform IL and AR simultaneously but is only capable of performing IL when a landmark is captured by the mobile device’s camera. An IL-AR method based on pedestrian tracking in subway stations was also proposed [11]. This system uses a smartphone to perform marker-based AR and hybrid IL using marker images and an inertial measurement unit (IMU). The challenge of this system is defining the marker location and the use of high-quality materials to minimize marker damage. Verde et al. [12] proposed an IL-AR architecture based on content delivery in museums. They implemented AR using Immersal AR and Easy AR and IL using Bluetooth Low Energy (BLE). The use of IL-AR was also proposed for use in navigation [13,14]. Both IL and AR were implemented using only a smartphone camera and 2-D visual markers [13]. Zhou et al. [14] used the ARCore software development kit (SDK) to implement AR and a combination of pedestrian dead reckoning (PDR) and BLE to implement IL. However, these IL-AR systems are mostly standalone systems and do not provide rich and secure context-aware information. Moreover, to the best of our knowledge, no IL-AR system supports two or more displaying modes and their associated user interactions at the same time.

ILARF primarily consists of three units: an indoor localization unit (ILU), a secure context-aware message exchange unit (SCAMEU), and an AR visualization and interaction unit (ARVIU). The ILU runs on a user device, such as a smartphone, to provide user location information in an indoor environment. The SCAMEU is responsible for exchanging context-aware information between ambient sensors and users. The unit also employs a web server to manage user profiles and settings. The ARVIU uses AR creation tools to display context-aware information at the appropriate screen positions on a mobile device, such as a smartphone or head-mounted display (HMD). This unit also processes interactions of users who use a smartphone or HMD. ILARF, thus, simultaneously supports the device screen displaying mode and the HMD displaying mode, as well as their associated user interactions.

The ILU uses device sensors and markers to determine the user’s location (or position). The device sensors, such as an accelerometer, gyroscope, and magnetometer, are used to determine the direction of the user. The markers are used as references to perform indoor localization [15]. The ILU employs two types of markers: invisible markers (e.g., Wi-Fi, BLE, and NFC signals) and visible markers (e.g., images and text). Indoor localization methods, regardless of whether they are inertial-based, fingerprint-based, multilateration-based, centroid-based, or marker-based, can all be adopted by the ILU to perform indoor localization.

The SCAMEU deals with data exchange between all units in ILARF via the ILARF server and the message queuing telemetry transport (MQTT) server [16]. The ILARF server provides the web connection service along with the database service. It is the core component of ILARF with regard to maintaining communication with user devices (e.g., smartphones) and managing user information (e.g., user profiles and settings). The MQTT server is used to publish context-aware information about the sensors and users. Possible sensor information includes the temperature, illuminance, and humidity, whereas possible user information includes the user’s location and personal profile. To make the communication of SCAMEU secure, a transport layer security (TLS) protocol [16] is employed to secure the exchanged data.

The ARVIU is responsible for displaying appropriate augmented information in the appropriate areas of a screen, and interacting with users to offer rich user experiences. This unit has three functions: context perception, information visualization, and user interaction. The context-perception function gathers contextual data from the surrounding environment to determine the augmented information to be displayed or visualized. The information visualization function then displays the augmented information with specific visualization effects overlaid atop appropriate images or appropriate areas of the screen. The user interaction function processes user interaction. ARVIU has two modes: device screen mode and HMD mode. In device screen mode, the user holds the device in hand and interacts with the system by pressing buttons shown on the device screen with visual information or effects overlaid on top of the view of the real scene. In HMD mode, the user wears an HMD device to perceive information atop scene views with immersive 3D effects. The user can interact with the system by gazing for a specific period of time at virtual buttons, gesturing at virtual buttons, and/or issuing voice commands.

Various methods that fit into ILARF will be discussed in a subsequent section. Using different methods can result in AR systems that offer different features. Based on ILARF, an AR system called Gym Augmented Reality (GAR) is developed as an Android app for use in gyms. Before using GAR, users must register their profiles and related information as GAR members. GAR can then help users locate equipment in a gym based on their workout programs and favorite exercise. For example, if a user wants to lose weight and likes to run, then GAR can show navigation information for reaching one of the available treadmills and show information about how to use it properly. Some gyms have course sessions such as yoga and dancing in dedicated rooms. For such cases, GAR can also guide the user to attend sessions that they are interested in. Furthermore, GAR also allows gym users to socialize with each other, which may help motivate them to go to the gym regularly and exercise consistently.

Numerous indoor AR systems have been proposed in the literature [17,18,19,20,21]. Of these systems, Endure [19], Climbing Gym [20], and Jarvis [21] are closely related to GAR. These three systems will be reviewed to understand today’s state-of-the-art AR systems for the use in gyms. These systems will be compared with GAR to demonstrate the superiority of GAR and show that using ILARF leads to the development of desirable indoor AR systems.

The contribution of this paper is five-fold. First, it proposes the ILARF framework that integrates IL and AR techniques to facilitate the development of indoor location-based AR systems. Second, ILARF provides rich and secure context-aware information exchanges. Third, ILARF simultaneously supports the device screen displaying mode and the HMD displaying mode, as well as their associated user interactions. Fourth, a practical AR system, GAR, to be used in gyms is developed based on ILARF. Fifth, GAR is compared with related AR systems to demonstrate its superiority, which in turn, will show that ILARF can indeed facilitate the development of desirable AR systems.

The rest of the paper is organized as follows. Section 2 presents an overview of ILARF and discusses schemes that can fit into the framework. Section 3 describes the implementation of GAR, which is developed on the basis of ILARF. GAR is compared with other related AR systems in Section 4. Finally, the paper is concluded in Section 5.

## 2. ILARF

**Figure 1:**
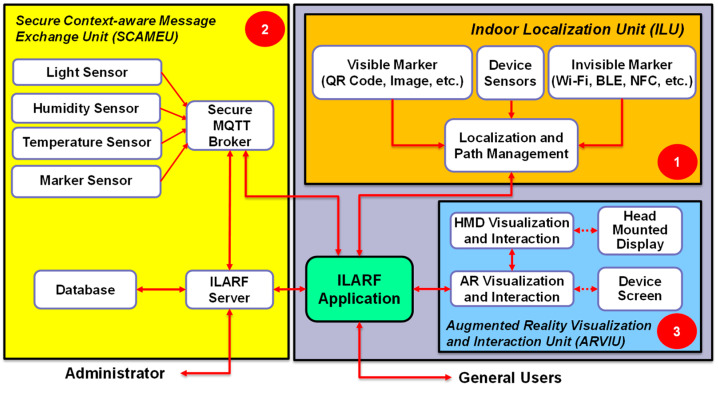
Diagram of ILARF architecture.

This section presents the ILARF architecture, possible technologies that fit into the architecture, and related background knowledge. Figure 1 depicts a diagram of the ILARF architecture, showing that it comprises three key units: ILU, SCAMEU, and ARVIU. The units are elaborated on below.

### 2.1. Indoor Localization Unit (ILU)

The ILU employs device sensors and markers to perform indoor localization. This subsection discusses some indoor localization methods that are suitable for the ILU. Generally speaking, the methods use various data, such as sensor readings, radiofrequency (RF) signals, and images, to perform IL in order to determine the location of the user device (UD) with or without the help of landmark devices (LD), which are deployed in advance at known locations. They are classified as inertia-based, fingerprint-based, multilateration-based, centroid-based, and marker-based, each of which is described below in a separate subsubsection.

#### 2.1.1. Inertia-Based IL Methods

Some UDs, such as mobile smartphones, are equipped with a variety of sensors. Certain sensors can be used to locate the UD. For example, the accelerometer, gyroscope, and magnetometer of a device can constitute an IMU and be used to determine the inertial state of the device [22]. An accelerometer is useful for determining the movements of a device. It provides 3D readings of acceleration in meters per second squared (m/s^2^) for the x, y, and z directions [23]. A gyroscope calculates the device’s angular movement in the x, y, and z directions. It can help determine the heading of a moving device using the measurement unit of radians per second (rad/s). The combination of an accelerometer and gyroscope is frequently used to determine the short-term location of a device. It can then determine the location of the user of the device, e.g., the location of a pedestrian. A magnetometer measures the value of the ambient geomagnetic field in the x, y, and z directions at a certain location. It helps reduce errors in the gyroscope readings by identifying a specific reference direction (usually North). Along with an accelerometer, a magnetometer can be used to determine a device’s rotational vector.

**Figure 2:**
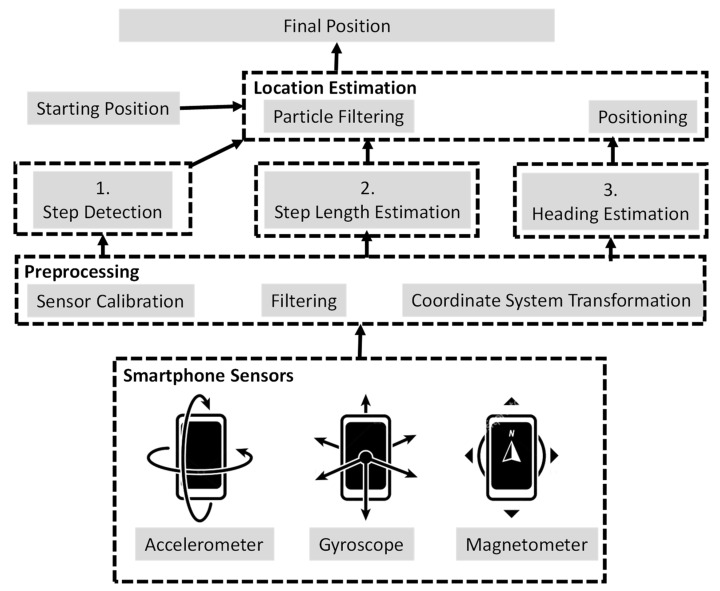
Illustration of the PDR-based IL method.

The localization method called pedestrian dead reckoning (PDR), which uses the above-mentioned IMU sensors, is suitable for an ILU. With IMU sensors, the PDR method estimates the step length and the heading of the device user or pedestrian. It can then obtain pedestrian-relative locations based on the previous position or a known position [24]. The advantage of the PDR method is that it has a low cost of installation and does not require extra UD sensors [25]. Figure 2 shows an illustration of the PDR IL method. Typically, a PDR system consists of three routines: (1) step detection, (2) step length estimation, and (3) heading estimation. IMU sensors can help the PDR method achieve a location accuracy of approximately several meters.

Particle filters [26] and zero-velocity detectors [27] are extensively used for step detection in PDR, but thresholding-based approaches are most commonly used in practice. Numerous algorithms are described in the literature that are based on thresholding using accelerometers [28,29], gyroscopes [30,31], or both. Furthermore, accelerometers, gyroscopes, and magnetometers can be combined as a sensor fusion value for step detection [32,33]. A step is assumed to be detected when the fusion value exceeds a predefined threshold or when a peak value is found in the time series of the fusion values. The above-mentioned methods are shown to be able to improve PDR localization accuracy.

#### 2.1.2. Fingerprint-Based IL Methods

Fingerprinting (or fingerprint-based) methods are widely used in IL. They are simple, easy to configure, and can localize a UD with high accuracy and without pricy hardware. Microsoft RADAR [34] is a famous Wi-Fi fingerprinting IL method that was proposed in 2000, and it has since been developed and improved by many researchers [35,36].

**Figure 3:**
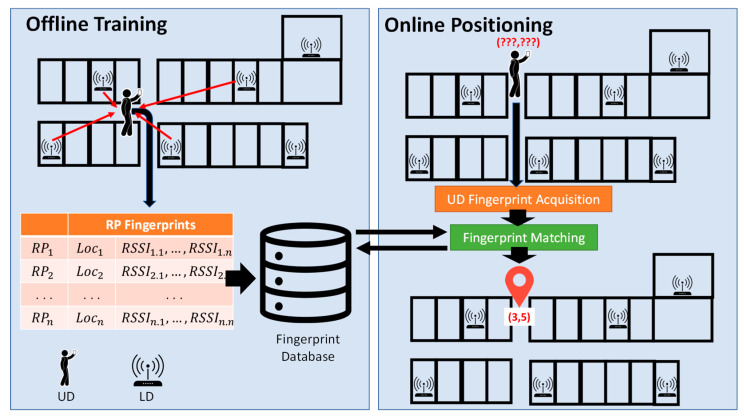
Illustration of the fingerprint-based IL method.

Figure 3 shows an illustration of a fingerprinting IL method [22] using LDs (e.g., Wi-Fi access points (AP)) deployed in advance at known locations to locate the UD. The fingerprinting IL method has two phases: offline training and online positioning, as shown in Figure 3. During the offline training phase, fingerprint data are collected and stored in a fingerprint database. Specifically, received signal strength indicator (RSSI) values are measured at indexed reference points (RPs) at fixed locations to create a radio map of the environment. Multiple signals of an LD are received and their RSSI values are measured and averaged at every RP. The RP’s location and the averaged RSSI value for every LD whose signals can be received at the RP are stored in the fingerprint database. In addition to original RSSIs, Gaussian models [37], histograms [38] of RSSIs, and other complex distributions [35] were also investigated to serve as the representations of RSSIs. Moreover, since RSSIs may vary in magnitude from one UD to another for the same location, other measurements or their derivatives were also used in place of RSSIs as fingerprints, namely, hyperbolic location fingerprinting (HLF) [39], the difference in phase [40], and the ordered RSSI [41]. The use of a Wi-Fi AP coverage area was reported to mitigate the impact of RSSI changes over time [42].

In the online positioning phase, a UD collects RSSIs from signals sent from various LDs to form a UD fingerprint. The UD fingerprint is then matched with those stored in the fingerprint databases to determine the UD location. Specifically, a matching process is employed to determine the similarity between the UD fingerprint and the RP fingerprints in the database according to various criteria, such as the Euclidean distance, Manhattan distance, and cross-correlation. The nearest-neighbor (NN) mechanism and its variants, including *k*-nearest neighbors (KNN) and weighted KNN mechanisms, are used to select RPs whose fingerprints are most similar to the UD’s [43]. Finally, the maximum likelihood estimator (MLE) [44], machine-learning techniques [45], and deep neural networks [46] can be applied to determine the UD location based on the selected RPs.

#### 2.1.3. Multilateration-Based IL Methods

The multilateration localization method is a geometric model primarily based on the e.g., information carried by received LD signals to determine the UD location. Its basic concept is described as follows. It first uses the strength measurements of signals sent from LDs (Wi-Fi APs) to estimate the distance between the UD and every LD. All the estimated distances are then employed to locate the UD. In this method, *n* (*n ≥* 3) LDs are deployed at specific locations for 2D IL. The relationship between the UD location and the *n* LD locations is formulated as:

(1) $$\begin{array}{c} {\left(x-x_{1}\right)}^{2}+{\left(y-y_{1}\right)}^{2}=d_{1}^{2}\\ {\left(x-x_{2}\right)}^{2}+{\left(y-y_{2}\right)}^{2}=d_{2}^{2}\\ \ldots \\ {\left(x-x_{n}\right)}^{2}+{\left(y-y_{n}\right)}^{2}=d_{n}^{2} \end{array}$$

**Figure 4:**
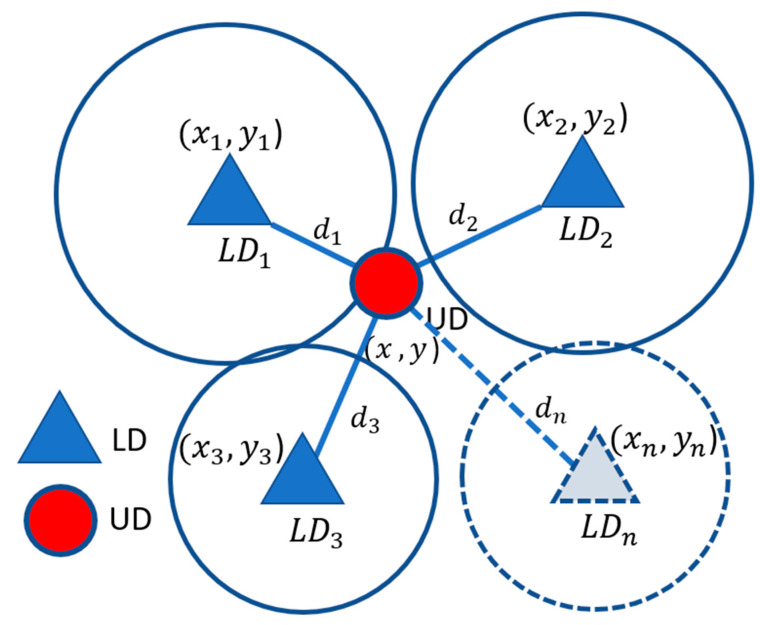
Illustration of the multilateration-based IL with *n* LDs.

Here, (x, y) are the location coordinates of the LD; $\left(x_{1},\;y_{1}\right),\;\left(x_{2},\;y_{2}\right)$, …, and $\left(x_{n},\;y_{n}\right)$ are the location coordinates of the *n* LDs; and $d_{1},d_{2}$, …, $d_{n}$ are, respectively, the distances from the UD to the *n* LDs. Figure 4 illustrates the multilateration method setting with three LDs. In practice, the distance *d* from the UD to an LD can be estimated by the Friis equation or other similar equations. The Friis equation is as follows:

(2) $$P_{r}=P_{t}\;G_{r}G_{t}{\left(\frac{\lambda }{4\pi d}\right)}^{2},$$

where $P_{r}$ is the signal power received by the receiver (i.e., the UD), $P_{r}$ is the transmitting power of the transmitter (i.e., the LD), $G_{r}$ is the gain of the receiver antenna, $G_{t}$ is the gain of the transmitter antenna, *λ* is the signal wavelength, and *d* is the distance between the transmitter and the receiver. Some studies assume the transmitting power $P_{r}$, the receiver antenna gain $G_{r}$, the transmitter antenna gain $G_{t}$, and the signal wavelength *λ* are fixed. The distance *d* is, thus, a function of the received signal power or strength and can be easily derived using RSSI values. With Equations (1) and (2) and the linear least-squares (LLS) mechanism, the location coordinates of the UD can then be obtained.

One problem of multilateration-based methods is that the distance estimation based on signal power (i.e., strength) is influenced by many factors, such as noise, multipath fading, shadowing effects, and the attenuation of signals [47]. Wang et al. [48] proposed a novel Wi-Fi-based scheme using curve fitting (CF) and location search techniques to construct a fitted RSSI distance function for each AP (i.e., LD), and Yang et al. [49] proposed preprocessing the RSSI raw data with a Gaussian filter to reduce the influence of measurement noise.

In addition to the mechanism that estimates the distance using signal power, there exist other distance-estimation mechanisms, such as time of arrival (TOA) [50] and time difference of arrival (TDOA) [51]. Moreover, the angle of arrival (AOA) [52] mechanism and its extended variants [53,54] use the angular relationship between the UD and every LD to calculate its location coordinates. Note that each of the above-mentioned mechanisms [46,47,48,49,50,51,52] are regarded as a multilateration-based IL method or one of its variants in this paper.

#### 2.1.4. Centroid-Based IL Methods

In centroid-based IL methods, the UD location can be estimated simply by the centroid of the locations of the detected LD. However, some centroid-based IL methods also employ RSSIs of different LDs to improve localization accuracy. For example, Subedi et al. [55] proposed a weighted centroid localization (WCL) method using BLE beacon devices. The method assigns a specific weight to a detected LD to calculate the weighted centroid (WC) based on the RSSI associated with the LD. Equations (3)–(5) are used to calculate the WC [55], which in turn, is regarded as the location of the UD:

(3) $$x_{w}=\frac{\sum_{i=1}^{n}x_{i}w_{i}}{\sum_{i=1}^{n}w_{i}}$$

(4) $$y_{w}=\frac{\sum_{i=1}^{n}y_{i}w_{i}}{\sum_{i=1}^{n}w_{i}}$$

(5) $$w_{i}=\frac{1}{d_{i}^{g}}$$

**Figure 5:**
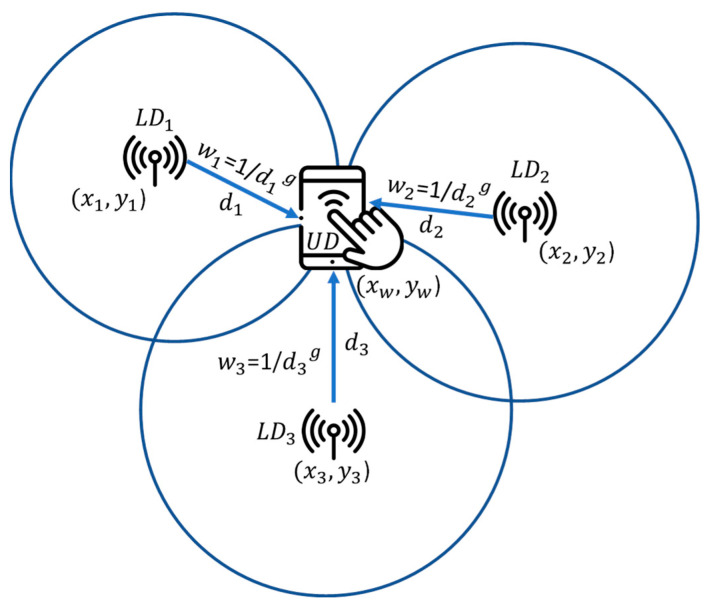
Illustration of the WCL method with three LDs.

In Equations (3)–(5), $\left(x_{w},\;y_{w}\right)$ are the location coordinates of the WC, $\left(x_{i},\;y_{i}\right)$ are the location coordinates of the *i*th detected LD, $d_{i}$ is the estimated distance between the UD and *i*th LD, g is the weighting, *n* is the total number of detected LDs, and 1≤i≤n. Similarly, $d_{i}$ can be derived from the Friis equation. Depending on how far apart the deployed LDs are, the weighting g can be set accordingly. A smaller value (closer to zero) of g corresponds to a WC approaching the geometrical centroid of the *n* detected LDs, whereas a greater value (e.g., 3) of g, corresponds to a WC closer to the LD with the strongest signal strength, as shown in [56]. Figure 5 illustrates the WCL method with three LDs.

#### 2.1.5. Marker-Based IL Methods

Marker-based IL methods [15,57] utilize markers as references to perform IL. Two major types of markers are used in such IL methods: invisible markers (e.g., Wi-Fi, BLE, and NFC signals) and visible markers (e.g., QR codes, images, and text) [15]. Note that the methods that use invisible markers to obtain the UD location can also be classified as fingerprint-based, multilateration-based, or centroid-based. Therefore, this paper mainly describes IL methods that use visible markers, such as Engfi-Gate [15] and Romli et al.’s method [57].

Engfi-Gate [15] uses invisible and visible markers to perform IL. BLE beacon packets sent by BLE beacon devices are used periodically as invisible markers and QR codes are used as visible markers. Engfi-Gate can determine the UD location using the WCL method when three or more BLE beacon devices are detected. Furthermore, when a QR code is recognized, its location information is decoded and the UD is assumed to have the same location as the QR code marker. Using both invisible and visible markers, Engfi-Gate can achieve sub-meter localization accuracy.

Romli et al. [57] developed a prototype AR mobile app for smart campus navigation within the library at their university. They took photos at key locations in the library to serve as visible markers. These photos or images were then registered to the Vuforia software to localize the UD and serve as triggers for pop-up AR augmented information or as AR objects when the UD detects registered photos or images.

### 2.2. Secure Context-Aware Message Exchange Unit

The SCAMEU handles context-aware data exchanges in ILARF. In order to collect and exchange context-aware data in real-time, a variety of devices, such as smartphones, tablets, wearable devices, smart bands, smart sensors, cameras, smart speakers, and GPS devices, can be connected using different protocols [58]. This subsection discusses four protocols suitable for SCAMEU to exchange the data of devices and users: the MQTT protocol [59,60], the Hypertext Transport Protocol (HTTP) [61,62], the Constrained Application Protocol (CoAP) [63,64], and the Advanced Message Queuing Protocol (AMQP) [65,66,67]. Moreover, the Transport Layer Security/Secure Socket Layer (TLS/SSL) protocol [68] is also discussed in the subsection for securing the exchanged data.

#### 2.2.1. MQTT

MQTT is a messaging protocol based on the publish–subscribe model. The first version of the MQTT protocol was introduced in 1999 [59]. MQTT v3.1 was released in 2013 and MQTT v5.0, announced in 2019, is the latest version. It was developed for resource-constrained devices with the aims of low cost, open-source, reliability, and simplicity [60].

**Figure 6:**
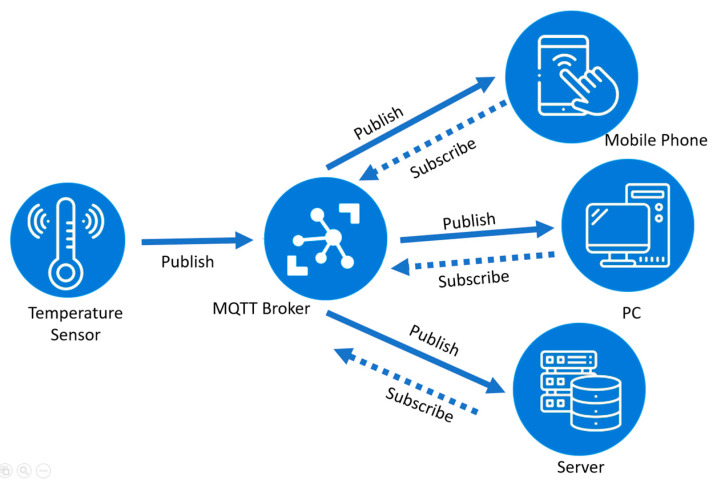
The MQTT publish–subscribe communication model.

The MQTT publish-subscribe model is depicted in Figure 6, where the MQTT broker (or server) is the center of the model. Depending on the implementation, a broker can simultaneously manage up to thousands of connected MQTT clients. Data are organized into a hierarchy of topics. A client can send subscribe messages to the broker to subscribe to different topics and can send publish messages to the broker to publish data for different topics. The broker is responsible for receiving and filtering every message, determining which clients have subscribed to the message, and sending the message to the subscribers.

For different application requirements, the MQTT protocol has three levels of quality of service (QoS): at-most-once, at-least-once, and exactly once. For the at-most-once service, the message is sent only once without acknowledgement. For the at-least-once service, two-way handshaking is employed and the message is resent by the sender several times until acknowledgement is received. For the exactly once service, the sender and the receiver use four-way handshaking to ensure only one copy of the message is received. MQTT transmits data based on the TCP/IP, which in turn, can be secured with the TLS/SSL protocol, as will be described later. A message can have a size of up to 256 MB and a header of 2 bytes according to the application. There are currently many MQTT platforms, such as Amazon Web Services, Microsoft Azure IoT, Adafruit, Facebook Messenger, and so on [61].

#### 2.2.2. HTTP

Tim Berners-Lee, a British scientist, initially proposed HTTP as a text-based online messaging protocol in 1989. The Internet Engineering Task Force (IETF) and the World Wide Web Consortium (W3C) then worked together to improve it. After several years of developing and improving HTTP, the IETF and W3C agreed to make it a standard protocol in 1996 [61].

HTTP supports the request–response model in client–server communication and uses a universal resource identifier (URI) to identify network resources. A client sends a message to a server requesting a resource with a specified URI. The server then sends back the resource associated with the specified URI to the client. HTTP is a text-based protocol and does not define the size of headers and message payloads. The default transport protocol for HTTP is TCP for connection-oriented communication, whereas TLS/SSL is used to ensure security. HTTP is a globally accepted web messaging standard that offers a variety of features such as persistent connections, request pipelining, and chunked transfer coding [61,62].

#### 2.2.3. CoAP

CoAP is a lightweight machine-to-machine (M2M) protocol from the IETF CoRE (Constrained RESTful Environments) Working Group [63,64]. This protocol supports both the request-response and the publish-subscribe models. CoAP was primarily created so that resource-constrained internet devices could interoperate.

Similar to HTTP, CoAP utilizes URI to identify resources. Unlike HTTP, CoAP is a binary protocol that has a header of 4 bytes along with short message payloads. CoAP employs the User Datagram Protocol (UDP) as its transport protocol for connectionless communication and uses Datagram Transport Layer Security (DTLS) to ensure security. CoAP offers two distinct degrees of QoS by employing “confirmable” and “non-confirmable” messages. Confirmable messages require receivers to acknowledge the messages, whereas non-confirmable messages do not.

CoAP has an extension to add a broker to offer publish–subscribe communication between subscribers and publishers. A subscriber sends a message with a URI to subscribe to a specific resource that is identified by the URI. When a publisher sends a message to update the information associated with the URI, the broker notifies all the subscribers that subscribed to the RUI of the updated information.

#### 2.2.4. AMQP

AMQP was created as a corporate messaging protocol for interoperability, provisioning, security, and dependability in 2003 [61]. Both the request-response and the publish–subscribe models are supported by AMQP [65]. It has many message-related capabilities, including topic-based publish-subscribe messaging, reliable queuing, flexible routing, and transaction [61].

In AMQP, either the publisher or the subscriber is required to build an “exchange” with a given name and to broadcast that name. By using the given name of this exchange, the publisher and the subscriber can find each other. After that, the subscriber creates a “queue” and attaches it to the exchange at the same time. The “binding” procedure is used to match received messages to a queue. AMQP is a binary protocol having several ways for exchanging messages: directly, in fanout form, by subject, and based on headers. AMQP has a header of 8 bytes and a short message payload of the maximum size depending on the programming technology [66,67]. Communication in AMQP is connection-oriented and TCP is used as the default transport protocol. Two QoS levels are provided by AMQP: the unsettle format (unreliable) and the settle format (reliable). Security in AMQP is provided via TLS/SSL or the Simple Authentication and Security Layer (SASL) protocol [65].

#### 2.2.5. TLS/SSL

SSL and TLS are both cryptographic protocols that encrypt and authenticate data transmitted between two entities, such as a web server and a web browser [68]. SSL was originally designed by Netscape in 1994 and became TLS in 1999. Since SSL is the predecessor of TLS, they are sometimes used interchangeably and referred to as TLS/SSL. TLS/SSL uses a four-way handshaking procedure based on a public-key cryptosystem such that the two communicating entities agree on a symmetric key to encrypt sensitive data in order to protect them.

MQTT, HTTP, and AMQP can apply TLS/SSL atop TCP for the purpose of ensuring data authentication, integrity, and confidentiality, whereas CoAP adopts DTLS atop UDP for the same purpose. The X.509 certificate can be used in TLS/SSL or DTLS to authenticate communicating entities in order to avoid many types of attacks, such as the man-in-the-middle attack, which can cause very significant damage. It is, thus, crucial to apply TLS/SSL to MQTT, HTTP, and AMQP and apply DTLS to CoAP.

### 2.3. AR Visualization and Interaction Unit

The ARVIU is responsible for displaying appropriate augmented information in appropriate areas of a device screen to offer rich user experiences and for user interaction. This unit has three functions: context perception, information visualization, and user interaction. The context-perception function gathers contextual data from the surrounding environment to determine the augmented information to be displayed or visualized. The information visualization function then displays the augmented information with specific visualization effects overlaid atop appropriate images or appropriate areas of the UD screen. The user interaction function captures the UD user’s actions from UD sensors (e.g., the touch screen, camera, and microphone), interprets the actions as commands, and then performs the corresponding routines.

ARVIU has two modes: device screen mode and HMD mode. The following section describes the two ARVIU modes.

#### 2.3.1. ARVIU Device Screen Mode

**Figure 7:**
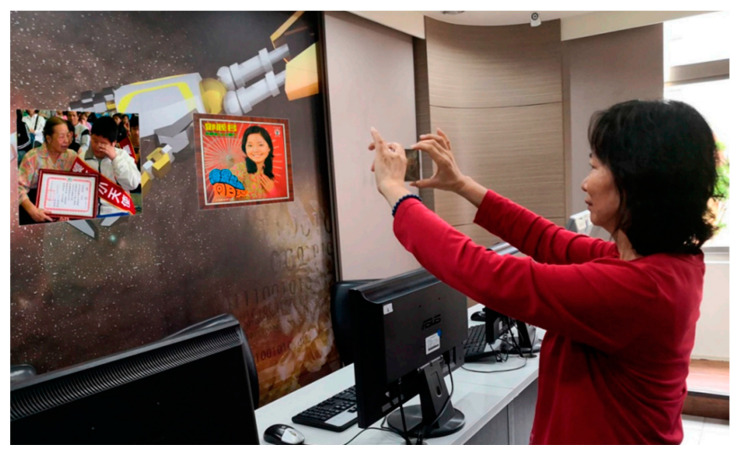
Demonstration of device screen mode (adapted from [69]).

In device screen mode, the 2D graphical user interface (GUI) and environmental images are displayed on the device screen. They are created with AR creation tools, e.g., the Google ARCore SDK and the Unity 3D game engine. Figure 7 shows an example of device screen mode, where context-aware information is displayed on the device screen and the user interacts with the system by tapping GUI buttons.

#### 2.3.2. ARVIU HMD Mode

**Figure 8:**
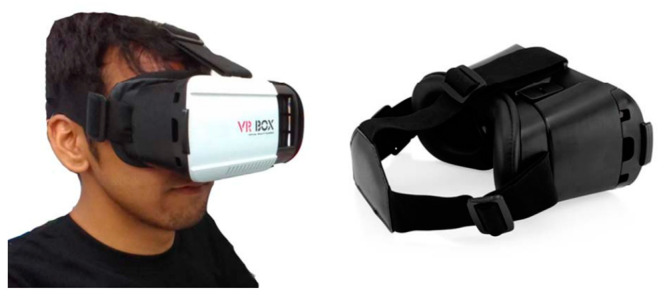
The HMD mode [15].

In HMD mode, the 3D GUI and environmental images are displayed on the device screen, which is divided into a left half and a right half. They are created with 3D AR creation tools, e.g., Google VR SDK, along with AR creation tools, e.g., Google ARCore SDK and the Unity 3D game engine. The left and right screen halves display images for the left and right eyes of the user, respectively. The two halves have slightly different images so that the user experiences the illusion of 3D. Figure 8 shows the HMD [15].

A binocular HMD, such as ASUS VIVE, or a head-mounted device in which a smartphone is embedded, such as the VR Box, is required for the HMD mode to induce a 3D immersive sensation in the user. When users wear a binocular HMD or insert their smartphones into head-mounted devices, they experience 3D objects with depth information. This is due to the fact that the 3D AR creation tool calculates the slightly different views required for each of the user’s eyes to produce the illusion of 3D. This mode employs virtual buttons to enable users to interact with the system because they are unable to tap the phone screen or the HMD screen. To interact with a virtual button, the user needs to place his or her finger in front of the camera and keep it stationary for a specific period of time. This is to simulate the finger remaining for a sufficient time over the virtual button to trigger the routine associated with the button. Alternatively, users can also interact with the system by hand gestures or voice commands.

## 3. Gym Augmented Reality

**Figure 9:**
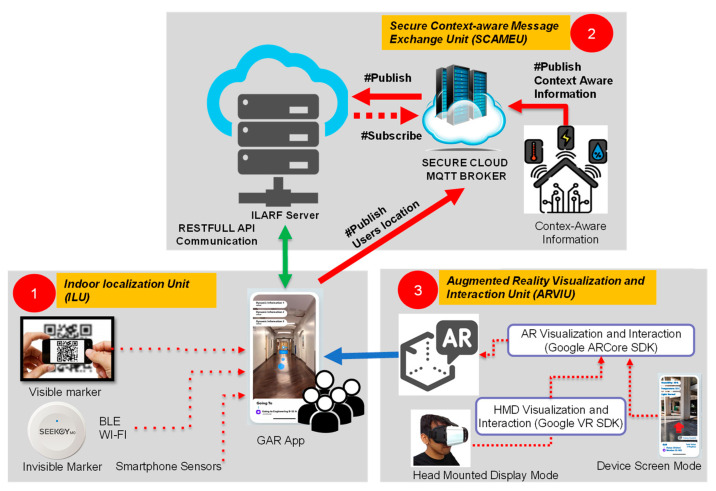
GAR implementation architecture.

ILARF is a framework that facilitates the development of AR systems. In this research, a prototype AR app to be used in gyms, Gym Augmented Reality (GAR), is developed based on ILARF. The implementation of the GAR prototype makes the following assumptions about the gym: there are four treadmills in Room 1, six stationary bicycles in Room 2, four barbell sets in Room 3, the yoga course in Room 4, and the dancing course in Room 5. Users can register their profiles and configure appropriate settings when they use GAR for the first time. GAR can then help the users locate gym equipment based on their workout programs or the favorite exercise listed in their profiles. For example, if a user wants to lose weight and likes to run, then the app will show the locations of available treadmills. For another example, if a gym has course sessions such as yoga and dancing in dedicated rooms, then GAR can also guide the user to attend sessions that they are interested in. It provides instructions on how to properly use the gym equipment and makes it possible for users to socialize with each other, which may help motivate them to go to the gym regularly. The GAR implementation architecture is illustrated in Figure 9.

### 3.1. Hardware and Software Specifications

This subsection describes the hardware and software requirements of GAR implementation. GAR is implemented on an Android-based UD and, in this paper, a Samsung Note 20 smartphone running on Android OS version 12, and supporting Bluetooth 4.0 was used. In the ILU, QR codes are used as the visible marker and BLE beacon signals (or messages) issued by Seekcy BLE devices are used as the invisible marker for determining the UD location and speed. The UD sensors, such as the accelerometer and the magnetometer, help obtain the UD direction. GAR uses the HTTP protocol for users to communicate with the ILARF server in the SCAMEU. The ILARF server is built on the basis of the Laravel PHP web server and the MySQL database. Some Raspberry Pi B+ devices, equipped with temperature, humidity, and light intensity sensors, are deployed in the areas surrounding the user to sense environmental data about the physical world. In order to enable context-aware communication between the sensors and ILARF server, the MQTT messaging protocol is employed. Furthermore, the Google ARCore SDK is used for AR creation in both device screen mode and HMD mode. Additionally, the Google VR SDK is employed in HMD mode.

### 3.2. ILU Implementation

Visible markers, invisible markers, UD sensors, and combinations of the first two are used in the ILU to determine the UD location. Below is a description of the ILU implementation.

#### 3.2.1. ILU Using Visible Markers

QR codes are used as the visible markers. GAR can determine the user’s location immediately by retrieving the location information encoded in the QR code once it is scanned by the UD camera and decoded successfully. Thus, only one QR code is needed to determine the UD location accurately. The localization accuracy depends on the distance from which a QR code image can be scanned and decoded successfully.

#### 3.2.2. ILU Using Invisible Markers

BLE beacon messages or signals sent by the BLE beacon devices are used as the invisible markers. The Seekcy BLE device is employed as the BLE beacon device. The location of the BLE beacon device is embedded into the BLE beacon messages. On receiving beacon messages sent by a beacon device, the UD can then determine the location of the beacon device. It can also measure the signal strengths of the beacon messages and estimate the UD location using the WCL method described earlier.

**Figure 10:**
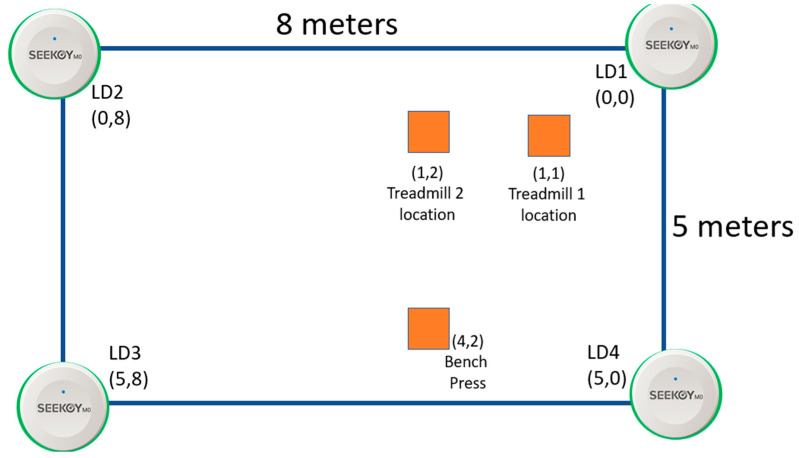
The locations of LDs and gym equipment in the experimental area coordinate system.

An experiment was set up to apply the WCL method in the ILU using invisible markers. The experimental setup is shown in Figure 10. Four LDs were placed in the corners of the 5 × 8 m experimental area. In addition, several gym equipment locations are predefined. The estimated UD location returned by the WCL method can be used to determine which gym kit is closest to the UD to help differentiate multiple kits. Moreover, the direction information provided by the method mentioned in the next sub-subsection, and the information derived from the QR code, if any, attached to the gym kit can also help differentiate multiple kits.

The UD speed can be calculated based on the history of the UD locations. By calculating the distance *d* between the current location and the previous location of the UD, the speed of the UD is calculated as *d*/*t*, where *t* is the time elapsed between the time associated with the previous location and the time associated with the current location.

#### 3.2.3. ILU Using UD Sensors

**Figure 11:**
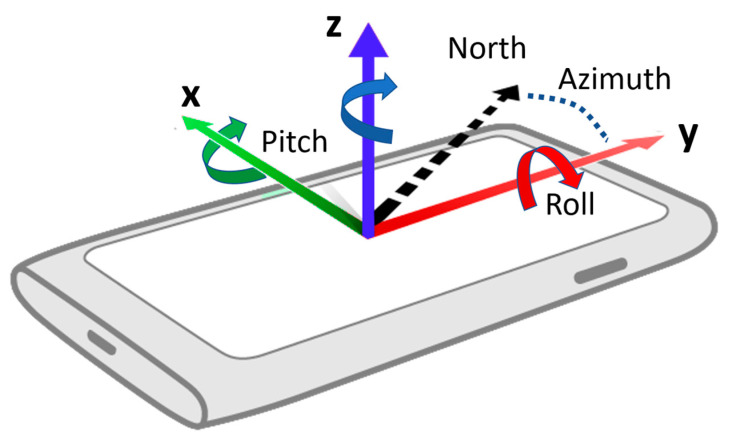
Illustration of the roll, pitch, and azimuth compared to the north direction.

UD sensors, such as the accelerometer and the magnetometer, are used in GAR ILU implementation to determine the UD direction or orientation. The UD direction is calculated via the steps described below. The first step is to obtain reading values from the accelerometer and the magnetometer. This can be done effectively by using the Android sensor API [70]. The second step is to use the reading values to calculate the UD direction of three components in radians. As shown in Figure 11, the three components are the azimuth, pitch, and roll, which are the rotation around the *z* axis, *x* axis, and *y* axis, respectively. The third step is to convert the azimuth from radians between 0 and 2 π to degrees between 0° and 360°. The UD direction can then be determined according to the azimuth in degrees. Specifically, the north is between 350° and 10°, the west is between 80° and 100°, and so on. Based on inertial sensors, the WalkCompass system [71] utilizes three modules, the human walk analysis, local walk direction estimator, and global walk direction estimator, to estimate the UD (i.e., smartphone) direction. The research in [71] conducted experiments for different users walking on various types of surfaces (e.g., carpet and tiled floor) with different holding positions of smartphones (e.g., portrait, landscape, and raising up for a call). It has shown that a median error of direction deviation of less than 8° can be achieved for the scenarios mentioned above. This demonstrates that using inertial sensors can be used to accurately estimate the direction of the UD for different environments. The ideas of the WalkCompass system can help GAR estimate UD direction for different environments.

#### 3.2.4. ILU Using Both QR Codes and Invisible Markers

GAR ILU combines IL methods using QR codes and invisible markers to perform localization calibration to improve localization accuracy. When a UD uses GAR and goes through the gym area, the WCL method using invisible markers (i.e., BLE beacon device signals) starts calculating the UD location. On receiving a signal from a BLE beacon device that acts as an LD, the UD can use the RSSI value to estimate the distance from itself to the beacon device according to the Friis equation, as shown in Equation (2).

In practice, the RSSI value of an LD signal is calculated according to:

(6) $$\mathrm{RSSI}=10\log\frac{P_{r}}{P_{ref}},$$

where the RSSI value is in decibel-milliwatts (dBm) and equal to 10 times the logarithmic value of the ratio of the received power $P_{r}$ to the reference power *P_ref_*, which is usually taken as 1.0 mW. Therefore, the power $P_{r}$ of the received signal can be derived from the RSSI value.

Putting $P_{r},P_{t\;},G_{r\;},\;\mathrm{and}\;G_{t}$ of the Friis equation in decibels (dB) (with *P_r_* and *P_t_* in dBm, and *G_r_* and *G_t_* in dBi) yields:

(7) $$P_{r\;}=P_{t\;}+G_{t\;}+G_{r}+20\log\left(\frac{\;\lambda }{4\pi d}\right)=P_{t\;}+G_{t\;}+G_{r}+20(\log\;\lambda -\log4-\log\pi -\log d),$$

where *P_t_*, *G_t_*, *G_r_*, and *λ* are often fixed and known in advance. Hence, the distance *d* between a UD and an LD can be derived using Equation (7).

Based on the simplified WCL method introduced earlier, a UD can determine its location *L* = (*L_x_*, *L_y_*) according to Equation (8) after receiving signals from *n* LDs, where (*L_x_*, *L_y_*) are the coordinates of the UD location *L* and *n* is usually taken as an integer greater than or equal to 3:

(8) $$L=\frac{\sum_{i=1}^{n}w_{i}L_{i}}{\sum_{i=1}^{n}w_{i}},$$

where *L_i_* represents the coordinates of the location of the *i*th LD, $w_{i}=\frac{1}{d_{i}}$; and $d_{i}$ is the estimated distance between the UD and the *i*th LD. As suggested in [72], $w_{i}=\frac{1}{d_{i}^{g}}$ can be set with g being a large value (e.g., 3) to weight longer distances marginally lower for the case when LDs have a long transmission range. However, g is set as 1 in the GAR ILU, and thus, $w_{i}=\frac{1}{d_{i}}$ in Equation (8).

As mentioned in [73], a UD can estimate the distance between itself and an LD when the WCL method is applied according to the RSSI value of the LD’s signal received on the basis of the Friis equation. This is under the assumption that the values of *P_t_*, *G_t_*, *G_r_*, and *λ* in the Friis equation are known. These values, however, are usually not known and are set to default values when the WCL method is applied. Moreover, even if the values are measured and set accordingly, they may change over time, owing to the instability of the signals and noise in the environment. Consequently, the values need to be calibrated if possible.

GAR performs WCL calibration based on the mechanism of the PINUS IL method [73], as described below. When a UD detects a QR code that is embedded with location information, the UD can take its location as the embedded location, which is a very accurate method. The UD can then calculate an accurate distance *d* between itself and an LD. By letting *C* represent the term ($P_{t\;}+G_{t}+G_{r}+20(\log\lambda -\log4-\log\pi ))$ in Equation (7), the following can be derived:

(9) $$P_{r\;}=C-20\;log\;d$$

(10) $$C=P_{r\;}+20\;log\;d$$

*P_r_* can be obtained by Equation (6). For a fixed and already known distance *d* (e.g., *d* = 1 m), the constant *C* can be calculated according to Equation (10) and regarded as the calibration constant associated with the LD. Afterwards, an unknown distance between the UD and the LD can then be estimated according to Equation (11); it is called the calibrated distance below.

(11) $$d=10^{\frac{C-P_{r}}{20}}$$

The UD can derive the calibration constant associated with every detected LD and store the constant locally in the UD to perform localization calibration in the future, as described below. When the UD receives signals from various LDs, it can use the RSSI value to derive *P_r_* based on Equation (6) and search locally for the calibration constant *C* associated with every LD to derive the calibrated distance between itself and every LD. With all the calibrated distances, the UD can derive more accurate weightings for all LDs. Consequently, the UD can derive its estimated location with higher accuracy based on Equation (8).

**Figure 12:**
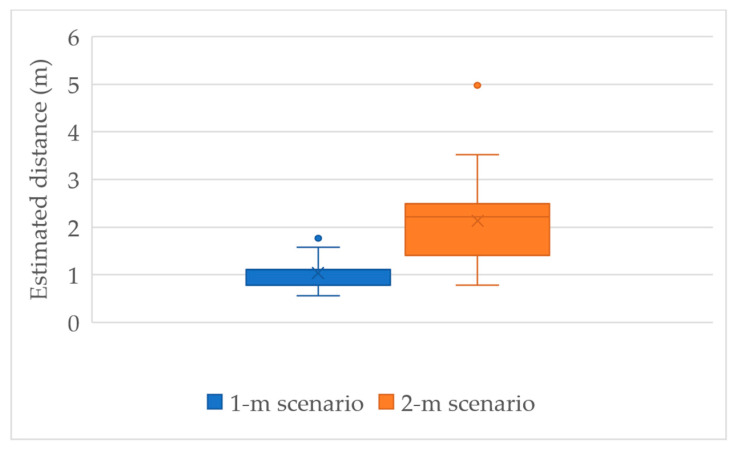
Box plot of the calibrated distance estimation.

This study has conducted experiments using the Samsung Note 20 smartphone as a UD and the Seekcy BLE device as an LD to estimate the calibrated distance according to Equation (11) for two scenarios. The first is the 1-m scenario where the UD and the LD are within a practical distance of 1 m, whereas the second is the 2-m scenario where the UD and the LD are within the practical distance of 2 m. The box plot in Figure 12 shows the calibrated distance estimation results of the two scenarios based on experimental data. There are 100 data pieces for either scenarios. Based on the box plot, the mean and the median of the estimated distances are very close to the practical distances. However, there still exist a few extreme values in the estimated results. Thus, it is suggested to gather a limited number (e.g., five) of estimated results and use their median to represent the final calibrated distance.

**Figure 13:**
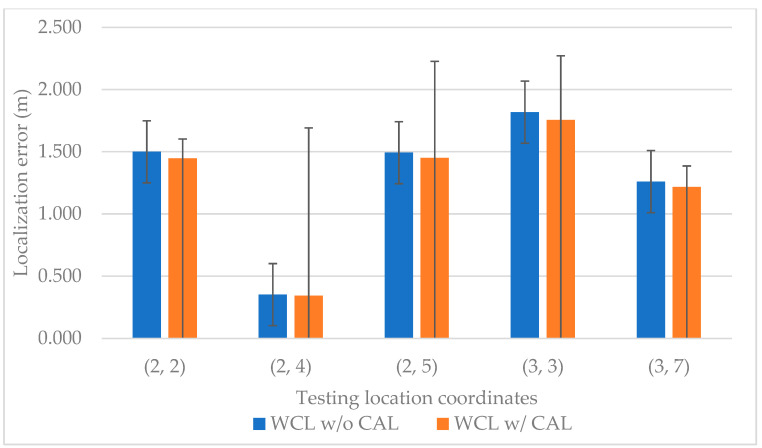
Localization error statistics of GAR WCL.

After the calibrated distance from the UD to every LD is obtained, the UD location can be determined by the WCL method. The localization error statistics of GAR WCL are shown in Figure 13. The *y* axis is the localization error and the *x* axis represents different testing location coordinates. The blue color represents the results of WCL using invisible markers without calibration (WCL w/o CAL), whereas the orange color represents WCL using invisible markers with QR code calibration (WCL w/CAL). Based on Figure 13, WCL w/CAL exhibits smaller localization errors than WCL w/o CAL.

### 3.3. SCAMEU Implementation

The GAR SCAMEU utilizes a secure cloud MQTT broker (or server) to exchange information from ambient sensors and user information to achieve context-awareness. This unit also employs an ILARF server, a PHP-based web server, to manage user profiles and settings.

#### 3.3.1. Secure Cloud MQTT Broker

**Figure 14:**
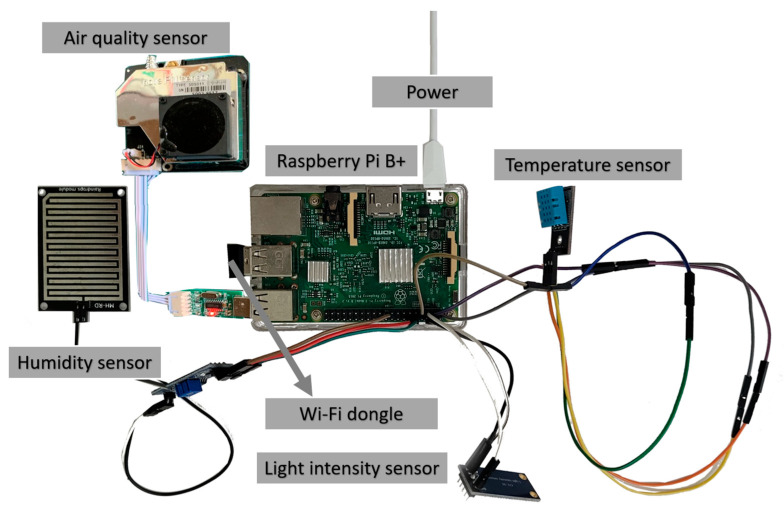
A Raspberry Pi B+ device equipped with Wi-Fi dongle USB and ambient sensors.

In the GAR SCAMEU implementation, ambient sensors such as temperature, light intensity, humidity, and air-quality sensors, are employed to sense the physical phenomena of the surrounding environment. The sensors are connected to a Raspberry Pi B+ device equipped with a Wi-Fi USB dongle, as shown in Figure 14. The sensed data are gathered by the Raspberry Pi B+ device, which acts as an MQTT publisher to embed sensed data into a packaged MQTT publisher message to be sent to the secure cloud MQTT broker. That is to say, the Raspberry Pi B+ device publishes the sensed data as context-aware information to the MQTT broker. Afterwards, the MQTT broker further publishes the sensed data to the ILARF server, which plays the role of an MQTT subscriber and, in turn, forwards the data to the UD. The implementation of the ILARF server will be described in Section 3.3.2. The implementation of the MQTT broker is described below.

The MQTT broker used by GAR is operated and maintained by the EMQX MQTT Cloud, which is the MQTT cloud service platform released by EMQ. It provides the service of accessing MQTT 5.0 as an all-in-one operation together with maintenance in a unique isolation environment. EMQX has built-in support for TLS/SSL that can ensure the security of transmission in data communication. The TLS/SSL implementation will be discussed in Section 3.3.3.

#### 3.3.2. ILARF Server

**Figure 15:**
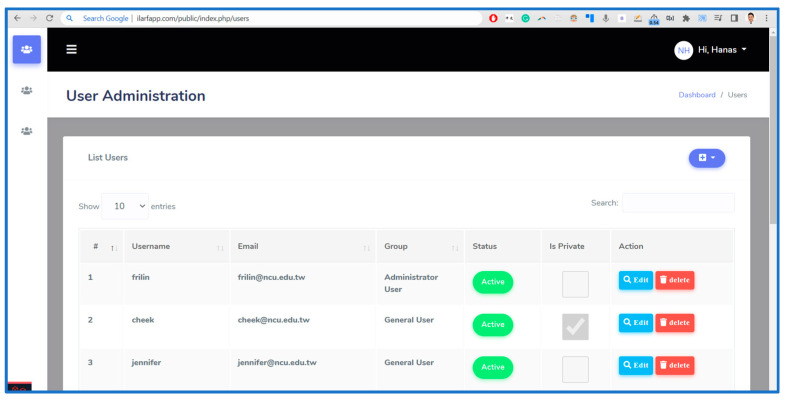
Screenshot of the ILARF server web interface.

The tasks of the ILARF server in the GAR SCAMEU implementation are to manage user profiles and settings and to forward data sent by the MQTT broker to the corresponding UD. The ILARF server is built based on the Laravel 8 PHP web server framework and the MySQL relational database management system. Before using GAR, users must register their information on the ILARF server. Two types of user roles are implemented in GAR: a general user who visits the gym and an administrator user who manages all users and the whole GAR system. A screenshot of the user administration management page of the ILARF server is shown in Figure 15.

#### 3.3.3. TLS/SSL Implementation

**Figure 16:**
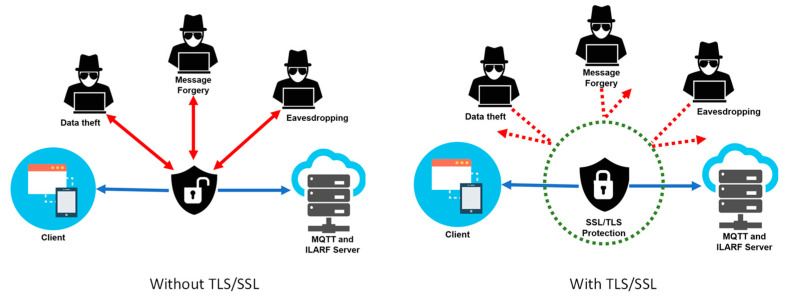
Data communication without (**left**) and with (**right**) TLS/SSL.

In the GAR SCAMEU implementation, both MQTT and HTTP protocols use TLS/SSL to ensure the security of the data communication. The data communication can be mainly classified as four types: (1) communication between the MQTT broker as the server and the Raspberry Pi B+ device as the client (publisher), (2) communication between the MQTT broker as the server and the device as the client (subscriber), (3) communication between the MQTT broker as the server and the ILARF server as the client (subscriber), and (4) communication between the ILARF server as the HTTP web server and the UD as the HTTP client. For all four types of data communication, messages sent between the server and the client are simple plain texts. Therefore, attackers can very easily tap into the communication, giving rise to the threats of data theft, data eavesdropping, message forgery, etc. To remove these threats, TLS/SSL is adopted combined with MQTT and HTTP to ensure data authentication, integrity, and confidentiality. Figure 16 shows a comparison between data communication without TLS/SSL and with TLS/SSL.

### 3.4. ARVIU Implementation

The ARVIU uses AR and VR SDKs to display context-aware information in the appropriate device screen areas and handle user interaction. There are two operation modes in the ARVIU: device screen mode and HMD mode. The following describes how GAR implements the two modes.

#### 3.4.1. GAR Device Screen Mode

**Figure 17:**
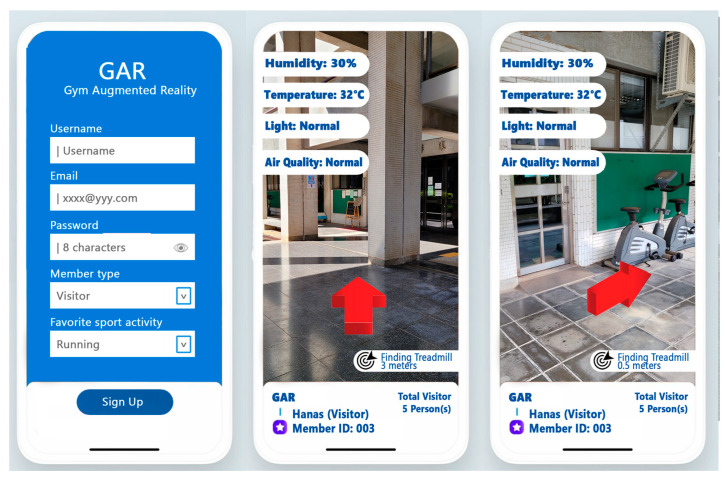
Device screen mode: screenshots of the registration page (**left**), main page when far from gym equipment (**middle**), and main page when near gym equipment (**right**).

The Unity3D game engine and the Google ARCore SDK are both used in GAR to synthesize scenes and display the 2D GUI on the phone screen. Figure 17 shows screenshots of the GAR GUI in device screen mode. The user interacts with GAR by tapping the buttons displayed on the GUI. Users can register their personal information or profile data on the ILARF server by filling in their username, email, password, member type, and their favorite sports activities when they use GAR for the first time. After registration, users can use GAR to move around a gym. The GAR ILU uses the BLE-based WCL method to locate a user who uses a UD running GAR. The ILU also uses UD sensors, such as the accelerometer and magnetometer, to determine the UD direction. Moreover, it performs localization calibration to improve the localization accuracy of WCL once the UD scans a QR code embedded with a known location. The UD location is used by GAR to show appropriate data from the sensors closest to the UD. Notably, the sensed data are published by the SCAMEU MQTT broker and sent to the ILARF server, which in turn forwards the sensed data to the UD according to the UD location and the locations of the sensors generating the data. Then, user-related information and context-aware information can be displayed at appropriate areas on the phone screen. The displayed information includes the humidity, temperature, light intensity, and air quality of the surrounding environment of the UD and the direction to the location of the user’s favorite sports activity or equipment, as well as the total number of visitors currently participating in the activity or using the equipment, as shown in Figure 17. In this mode, users can interact with the system by tapping buttons shown on the screen.

#### 3.4.2. GAR HMD Mode

**Figure 18:**
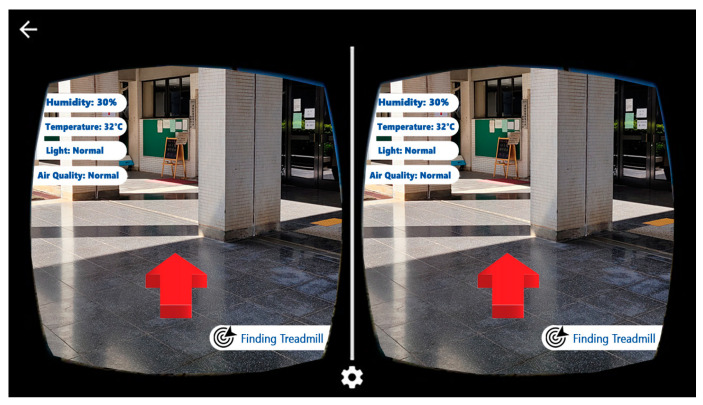
Screenshot of GAR HMD mode. (The left arrow in the upper left corner indicates the “EXIT” button to exit the app).

In addition to the Unity 3D game engine and the Google ARCore SDK, the Google VR SDK is used in GAR HMD mode to split the phone screen into left and right halves for the left and right eyes of the user, respectively. Figure 18 shows a screenshot of GAR HMD mode. A head-mounted device, such as the VR Box, is required to use this mode. The user inserts their smartphone into the head-mounted device to experience the sensation of seeing 3D objects with depth information. This is because the Google VR SDK calculates the slightly different views required for each eye to produce the illusion of 3D. In GAR HDM mode, user-related information and context-aware information are displayed. The displayed information includes the humidity, temperature, and light intensity of the surrounding environment of the user, as well as the direction of the location of the user’s favorite sports activity or equipment. This mode employs virtual buttons for users to interact with as they are unable to tap the screen of their phone once it is placed within the head-mounted device. The user needs to place their finger in front of the camera and keep it stationary for a specific period of time to simulate their finger pressing a virtual button to trigger specific routines.

## 4. Comparison of GAR and Related Systems

GAR is built based on the ILARF framework. To show that ILARF can indeed facilitate the development of desirable AR systems with multiple advantages, three related gym AR/VR (virtual reality) systems are reviewed, namely, Endure [19], Climbing Gym [20], and Jarvis [21], as described in Section 4.1. After that, GAR is compared with these related systems in Section 4.2.

### 4.1. Overview of Related Systems

**Figure 19:**
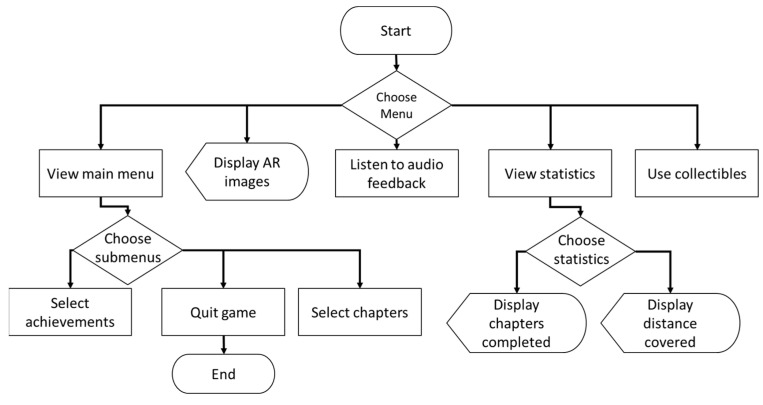
Flowchart of the Endure system.

Endure [19] is an AR-based fitness application that incorporates gaming with gym activities. A flowchart for Endure is shown in Figure 19. Its purpose is to increase user motivation to do more exercise, and it can be used indoors or outdoors. It does not offer indoor localization but does employ the global positioning system (GPS) outdoors to calculate user travel distance. The calculated distance is logged daily and shown on the phone screen for the user to track their exercise progress.

**Figure 20:**
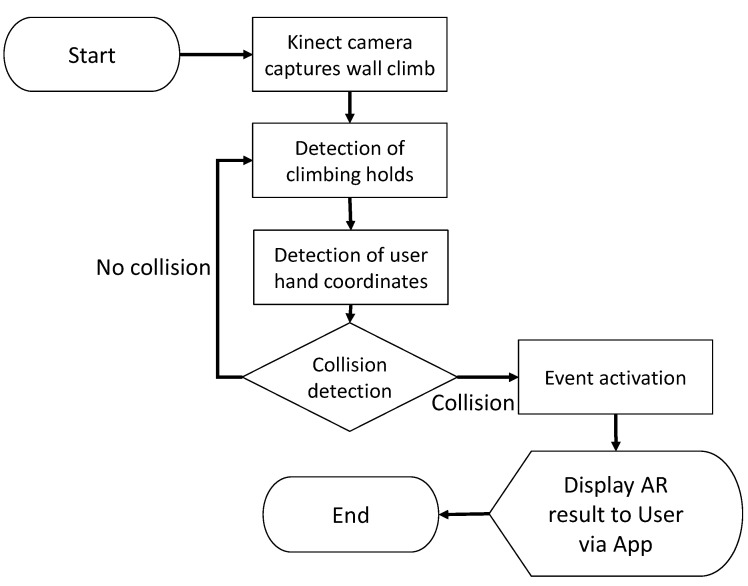
Flowchart for the Climbing Gym system.

Climbing Gym [20] is an AR application for teaching indoor climbing techniques via AR visualization. A flowchart for Climbing Gym can be seen in Figure 20. Initially, Climbing Gym acquires a climbing wall image using the Kinect ONE v2 RGB video camera and then preprocesses it. After that, image detection is used to obtain the coordinates of the climbing holds. Climbing Gym uses an HTTP connection to exchange messages to transmit the coordinates of detected climbing holds between the web server and the application. Climbing holds that form a route to climb are then selected and visualized onto the climbing wall by a projector or onto the phone screen for various training programs customized for different users.

**Figure 21:**
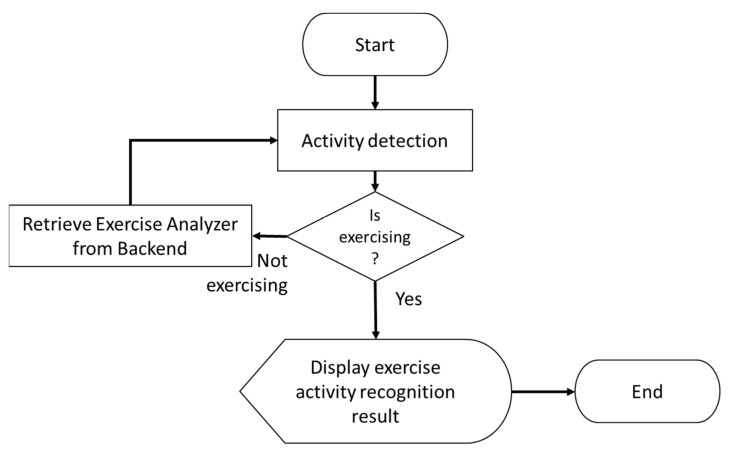
Flowchart of the Jarvis system.

Jarvis [21] is a VR application that runs as a virtual fitness assistant. A flowchart of the Jarvis system is shown in Figure 21. It uses a 3D GUI with an HMD to provide the users with an immersive and engaging workout experience in the gym. Miniature sensors are attached to exercise machines to send sensed data via BLE communication to track exercise information such as exercise type, repetition counts, etc. Jarvis relies on the tracked exercise information to derive and show the best way to perform the exercise for users to achieve their exercise goals (e.g., improving a target muscle group).

### 4.2. Comparison of GAR and Related Systems

Table 1 shows the comparison results by which it can be observed that GAR has some advantages over the related systems, as described below. First, GAR provides indoor localization information, whereas the others do not. GAR employs an invisible marker (i.e., the BLE beacon signal), a visible marker (i.e., the QR code), and UD sensors (i.e., the accelerometer and magnetometer) to provide accurate UD location and direction information. Suitable and useful information can then be derived accordingly and be displayed on appropriate areas of the screen. The accurate location and direction information are also useful for GAR to navigate users to certain points of interest.

Second, GAR is more secure than the other systems and the privacy of GAR users is, thus, highly protected. GAR uses TLS/SSL to secure MQTT and HTTP to ensure data authentication, integrity, and confidentiality. In contrast, no other systems consider the security aspect of data communication. Therefore, they are more vulnerable to cyberattacks, loss of user privacy, and leaks.

Third, GAR has richer context-aware information than the other systems. The information provided by GAR includes the data from ambient sensors and the public profile data of nearby users. In contrast, only limited information is provided by the other systems. For example, only the travel distance, the climbing holds of a climbing route, and sensor data are displayed in Endure, Climbing Gym, and Jarvis, respectively.

Fourth, Endure and Climbing Gym use a 2D GUI, whereas Jarvis and GAR provide a 3D GUI and the HMD mode. Compared to the 2D GUI, the 3D GUI provides more intuitive forms of user interaction. Moreover, the projector visualization of Jarvis and the 3D GUI in the HMD mode of Jarvis and GAR also give users additional hands-free interaction experiences.

The limitations of GAR implemented based on ILARF are that GAR is still a prototype and that its ILU is realized in a room instead of a practical gym environment. Furthermore, ILU, SCAMEU, and ARVIU have only partial functions specified in ILARF. Despite these limitations, GAR is shown to be comparably good when compared with related systems, which in turn, demonstrates that ILARF is a good framework.

## 5. Conclusions

This paper proposed an indoor location-based augmented reality framework (ILARF) for developing superior indoor augmented-reality (AR) systems. ILARF has three major units: the indoor localization unit (ILU), the secure context-aware message exchange unit (SCAMEU), and the AR visualization and interaction unit (ARVIU). The ILU runs on a mobile device such as a smartphone and utilizes visible markers, invisible markers, and device sensors to localize the user device (UD). The SCAMEU utilizes a MQTT server for clients to subscribe and publish data, such as ambient sensor data and user information, for context-awareness. It also employs a web server to manage user profiles and settings via the HTTP protocol. ARVIU uses AR/VR creation tools to display context-aware information in appropriate areas of the device screen. It has two modes: device screen mode and HMD mode, both of which have their own manner of user interactions.

In summary, the novelties of ILARF are as follows. First, it combines IL and AR techniques to facilitate the development of indoor location-based AR systems. Second, it adopts the MQTT, HTTP, and TLS protocols to provide users with rich and secure context-aware information exchanges. Third, ILARF uses AR/VR creation tools to easily support both device screen mode and HMD mode, as well as their associated user interactions, at the same time.

To show that ILARF can indeed facilitate developers to create novel and superior indoor location-based AR systems, one prototype AR app for gyms, Gym Augmented Reality (GAR), is developed based on ILARF. Users can register their profiles and configure the settings when they use GAR for the first time. Afterwards, GAR helps users locate gym equipment based on their workout programs or favorite exercise by showing directions and also provides instructions on how to properly use the gym equipment. Furthermore, GAR also enables users to socialize with each other, which may help motivate them to go to the gym regularly. GAR is compared with other related systems, namely, Endure, Climbing Gym, and Jarvis, to show that it is superior by virtue of ILARF. In addition to gyms, ILARF can be used to realize AR systems in other domains, for example in classes, factories, warehouses, farms, kitchens, museums, etc. In practice, ILARF can be applied to environments that require visualization using AR, IL, and data communication at the same time. Typical environments include the smart manufacturing [74], smart city [75], smart home [76], and smart campus [77], etc.

Currently, GAR is still in prototype form and, thus, has some limitations. Its ILU was implemented in a room instead of a real gym environment. Moreover, ILU, SCAMEU, and ARVIU have only partial functions specified in ILARF. Despite these limitations, GAR was shown to be better than related AR systems. In the future, more functionality for GAR is planned to be added. The scope for future improvement includes the interaction and information provided for users. For example, a path planning function will be created to construct paths for users to go to specific locations of interest. A function for showing 3D views of exercise moves so that users can receive information about how to exercise properly and efficiently is also planned. Moreover, more novel AR systems based on ILARF will be developed. Specifically, an AR system for smart classes and an AR system for smart factories are both planned.

## Figures and Tables

**Table 1:** Comparison of GAR and related systems.

	Systems	Endure [19]	Climbing Gym [20]	Jarvis [21]	GAR
Properties					
Indoor localization		No	No	No	BLE Beacon Signals + QR Code + UD Sensors
Secure message exchange		No	No	No	TLS/SSL for MQTT and HTTP
Context-aware Information		Travel Distance	Climbing Holds	Sensor Data	Sensor Data + Nearby User Profiles
AR Interface		2D GUI	2D GUI	3D GUI + HMD	2D GUI + 3D GUI + HMD

## Data Availability

Not applicable.

## References

[1] Zafari F., Gkelias A., Leung K.K. (2019). A survey of indoor localization systems and technologies. IEEE Commun. Surv. Tutor..

[2] Sirohi P., Agarwal A., Maheshwari P. A survey on augmented virtual reality: Applications and future directions. Proceedings of the 2020 Seventh International Conference on Information Technology Trends (ITT).

[3] El-Sheimy N., Li Y. (2021). Indoor navigation: State of the art and future trends. Satell. Navig..

[4] Vy T., Nguyen T., Shin Y. Pedestrian indoor localization and tracking using hybrid Wi-Fi/PDR for iPhones. Proceedings of the 2021 IEEE 93rd Vehicular Technology Conference (VTC2021-Spring).

[5] Shamsfakhr F., Antonucci A., Palopoli L., Macii D., Fontanelli D. (2022). Indoor localization uncertainty control based on wireless ranging for Robots Path Planning. IEEE Trans. Instrum. Meas..

[6] Ma W., Zhang S., Huang J. (2021). Mobile augmented reality based indoor map for improving geo-visualization. PeerJ Comput. Sci..

[7] de Souza Cardoso L.F., Mariano FC M.Q., Zorzal E.R. (2020). A survey of industrial augmented reality. Comput. Ind. Eng..

[8] Siriwardhana Y., Porambage P., Liyanage M., Ylianttila M. (2021). A survey on mobile augmented reality with 5G mobile edge computing: Architectures, applications, and technical aspects. IEEE Commun. Surv. Tutor..

[9] Baek F., Ha I., Kim H. (2019). Augmented reality system for facility management using image-based indoor localization. Autom. Constr..

[10] An H.W., Moon N. (2021). Indoor positioning system using pyramidal beacon in mobile augmented reality. Advances in Computer Science and Ubiquitous Computing.

[11] Lee G., Kim H. (2020). A hybrid marker-based indoor positioning system for pedestrian tracking in subway stations. Appl. Sci..

[12] Verde D., Romero L., Faria P.M., Paiva S. Architecture for museums location-based content delivery using augmented reality and beacons. Proceedings of the 2022 IEEE International Smart Cities Conference (ISC2).

[13] Martin A., Cheriyan J., Ganesh J.J., Sebastian J., Jayakrishna V. (2021). Indoor navigation using augmented reality. EAI Endorsed Trans. Creat. Technol..

[14] Zhou B., Gu Z., Ma W., Liu X. Integrated BLE and PDR indoor localization for geo-visualization mobile augmented reality. Proceedings of the 2020 16th IEEE International Conference on Control, Automation, Robotics and Vision (ICARCV) 2020.

[15] Subakti H., Jiang J.-R. A marker-based cyber-physical augmented-reality indoor guidance system for smart campuses. Proceedings of the 2016 IEEE 14th International Conference on Smart City (SmartCity).

[16] Chen F., Huo Y., Zhu J., Fan D. A review on the study on MQTT security challenge. Proceedings of the 2020 IEEE International Conference on Smart Cloud (SmartCloud).

[17] Sureshkumar S., Agash C.P., Ramya S., Kaviyaraj R., Elanchezhiyan S. Augmented Reality with Internet of Things. Proceedings of the 2021 International Conference on Artificial Intelligence and Smart Systems (ICAIS).

[18] Tayef S.H., Rahman M.M., Sakib M.A.B. Design and Implementation of IoT based Smart Home Automation System. Proceedings of the 2021 24th International Conference on Computer and Information Technology (ICCIT).

[19] Nair YN I., Azman F., Rahim F.A., Cheng L.K. Endure: Augmented reality fitness mobile application. Proceedings of the 2019 IEEE 4th International Conference on Computer and Communication Systems (ICCCS).

[20] Gurieva N., Guryev I., Pacheco Sánchez R., Salazar Martínez E. (2019). Augmented reality for personalized learning technique: Climbing gym case study. Open J. Inf. Technol..

[21] Rabbi F., Park T., Fang B., Zhang M., Lee Y. (2018). When virtual reality meets Internet of things in the gym: Enabling immersive interactive machine exercises. Proc. ACM Interact. Mob. Wearable Ubiquitous Technol..

[22] Ashraf I., Hur S., Park Y. (2020). Smartphone sensor based indoor positioning: Current status, opportunities, and future challenges. Electronics.

[23] Zhao H., Cheng W., Yang N., Qiu S., Wang Z., Wang J. (2019). Smartphone-based 3D indoor pedestrian positioning through multi-modal data fusion. Sensors.

[24] Park S., Lee J.H., Park C.G. (2021). Robust pedestrian dead reckoning for multiple poses in smartphones. IEEE Access.

[25] Wang Q., Luo H., Xiong H., Men A., Zhao F., Xia M., Ou C. (2020). Pedestrian dead reckoning based on walking pattern recognition and online magnetic fingerprint trajectory calibration. IEEE Internet Things J..

[26] Pinchin J., Hide C., Moore T. A particle filter approach to indoor navigation using a foot mounted inertial navigation system and heuristic heading information. Proceedings of the 2012 International Conference on Indoor Positioning and Indoor Navigation (IPIN).

[27] Skog I., Nilsson J.O., Händel P. Evaluation of zero-velocity detectors for foot-mounted inertial navigation systems. Proceedings of the 2010 International Conference on Indoor Positioning and Indoor Navigation.

[28] Castaneda N., Lamy-Perbal S. An improved shoe-mounted inertial navigation system. Proceedings of the 2010 International Conference on Indoor Positioning and Indoor Navigation.

[29] Nilsson J.O., Gupta A.K., Händel P. Foot-mounted inertial navigation made easy. Proceedings of the 2014 International Conference on Indoor Positioning and Indoor Navigation (IPIN).

[30] Feliz Alonso R., Zalama Casanova E., Gómez García-Bermejo J. (2009). Pedestrian tracking using inertial sensors. Phys. Agents.

[31] Woodman O., Harle R. Pedestrian localisation for indoor environments. Proceedings of the 10th International Conference on Ubiquitous Computing.

[32] Qian J., Ma J., Ying R., Liu P., Pei L. An improved indoor localization method using smartphone inertial sensors. Proceedings of the International Conference on Indoor Positioning and Indoor Navigation.

[33] Bird J., Arden D. (2011). Indoor navigation with foot-mounted strapdown inertial navigation and magnetic sensors [emerging opportunities for localization and tracking]. IEEE Wirel. Commun..

[34] Bahl P., Padmanabhan V.N. RADAR: An in-building RF-based user location and tracking system. Proceedings of the IEEE INFOCOM 2000.

[35] Youssef M., Agrawala A. The Horus WLAN location determination system. Proceedings of the 3rd International Conference on Mobile Systems, Applications, and Services.

[36] Kilinc C., Mostafa SA M., Islam R.U., Shahzad K., Andersson K. Indoor taxi-cab: Real-time indoor positioning and location-based services with ekahau and android OS. Proceedings of the 2014 Eighth International Conference on Innovative Mobile and Internet Services in Ubiquitous Computing.

[37] Haeberlen A., Flannery E., Ladd A.M., Rudys A., Wallach D.S., Kavraki L.E. Practical robust localization over large-scale 802.11 wireless networks. Proceedings of the 10th Annual International Conference on Mobile Computing and Networking.

[38] Roos T., Myllymäki P., Tirri H., Misikangas P., Sievänen J. (2002). A probabilistic approach to WLAN user location estimation. Int. J. Wirel. Inf. Netw..

[39] Kjærgaard M.B. (2011). Indoor location fingerprinting with heterogeneous clients. Pervasive Mob. Comput..

[40] Dong F., Chen Y., Liu J., Ning Q., Piao S.A. calibration-free localization solution for handling signal strength variance. Proceedings of the International Workshop on Mobile Entity Localization and Tracking in GPS-Less Environments.

[41] Yedavalli K., Krishnamachari B., Ravula S., Srinivasan B. Ecolocation: A sequence based technique for RF localization in wireless sensor networks. Proceedings of the Fourth International Symposium on Information Processing in Sensor Networks (IPSN).

[42] Ashraf I., Hur S., Park Y. (2019). Indoor positioning on disparate commercial smartphones using Wi-Fi access points coverage area. Sensors.

[43] Yigit H. A weighting approach for KNN classifier. Proceedings of the 2013 International Conference On Electronics, Computer and Computation (ICECCO).

[44] Bialer O., Raphaeli D., Weiss A.J. (2013). Maximum-likelihood direct position estimation in dense multipath. IEEE Trans. Veh. Technol..

[45] Li Y., Gao Z., He Z., Zhuang Y., Radi A., Chen R., El-Sheimy N. (2019). Wireless fingerprinting uncertainty prediction based on machine learning. Sensors.

[46] Zhang W., Liu K., Zhang W., Zhang Y., Gu J. (2016). Deep neural networks for wireless localization in indoor and outdoor environments. Neurocomputing.

[47] Zhu X., Feng Y. (2013). RSSI-based algorithm for indoor localization. Commun. Netw..

[48] Wang B., Zhou S., Liu W., Mo Y. (2014). Indoor localization based on curve fitting and location search using received signal strength. IEEE Trans. Ind. Electron..

[49] Yang B., Guo L., Guo R., Zhao M., Zhao T. (2020). A novel trilateration algorithm for RSSI-based indoor localization. IEEE Sens. J..

[50] Chen X., Song S., Xing J. A ToA/IMU indoor positioning system by extended Kalman filter, particle filter and MAP algorithms. Proceedings of the 2016 IEEE 27th Annual International Symposium on Personal, Indoor, and Mobile Radio Communications (PIMRC).

[51] Gentner C., Jost T. Indoor positioning using time difference of arrival between multipath components. Proceedings of the 2013 International Conference on Indoor Positioning and Indoor Navigation (IPIN).

[52] Malajner M., Planinsic P., Gleich D. (2011). Angle of arrival estimation using RSSI and omnidirectional rotatable antennas. IEEE Sens. J..

[53] Chen Y.M., Tsai C.L., Fang R.W. TDOA/FDOA mobile target localization and tracking with adaptive extended Kalman filter. Proceedings of the 2017 International Conference on Control, Artificial Intelligence, Robotics & Optimization (ICCAIRO).

[54] Yen H.C., Yang L.Y.O., Tsai Z.M. (2022). 3-D Indoor Localization and Identification Through RSSI-Based Angle of Arrival Estimation with Real Wi-Fi Signals. IEEE Trans. Microw. Theory Tech..

[55] Subedi S., Kwon G.R., Shin S., Hwang S.S., Pyun J.Y. Beacon based indoor positioning system using weighted centroid localization approach. Proceedings of the 2016 Eighth International Conference on Ubiquitous and Future Networks (ICUFN) 2016.

[56] Subedi S., Pyun J.-Y. (2020). A survey of smartphone-based indoor positioning system using RF-based Wireless Technologies. Sensors.

[57] Romli R., Razali A.F., Ghazali N.H., Hanin N.A., Ibrahim S.Z. (2019). Mobile augmented reality (AR) marker-based for indoor library navigation. IOP Conference Series: Materials Science and Engineering.

[58] Manaligod H.J., Diño M.J., Ghose S., Han J. (2019). Context computing for internet of things. J. Ambient Intell. Humaniz. Comput..

[59] Bandyopadhyay S., Bhattacharyya A. Lightweight Internet protocols for web enablement of sensors using constrained gateway devices. Proceedings of the 2013 International Conference on Computing, Networking and Communications (ICNC).

[60] Stanford-Clark A., Truong H.L. (2013). Mqtt for sensor networks (mqtt-sn) protocol specification. Int. Bus. Mach. (IBM) Corp. Version.

[61] Naik N. Choice of effective messaging protocols for IoT systems: MQTT, CoAP, AMQP and HTTP. Proceedings of the 2017 IEEE International Systems Engineering Symposium (ISSE).

[62] Naik N., Jenkins P., Davies P., Newell D. Native web communication protocols and their effects on the performance of web services and systems. Proceedings of the 2016 IEEE International Conference on Computer and Information Technology (CIT).

[63] Thangavel D., Ma X., Valera A., Tan H.-X., Tan C.K.-Y. Performance evaluation of MQTT and CoAP via a common middleware. Proceedings of the 2014 IEEE Ninth International Conference on Intelligent Sensors, Sensor Networks and Information Processing (ISSNIP).

[64] Ludovici A., Moreno P., Calveras A. (2013). TinyCoAP: A novel constrained application protocol (CoAP) implementation for embedding restful web services in wireless sensor networks based on TinyOS. J. Sens. Actuator Netw..

[65] Han N.S. (2015). Semantic service provisioning for 6LoWPAN: Powering internet of things applications on Web. Ph.D. Thesis.

[66] Marsh G., Sampat A.P., Potluri S., Panda D.K. (2008). Scaling Advanced Message Queuing Protocol (AMQP) Architecture with Broker Federation and Infiniband.

[67] Luzuriaga J.E., Perez M., Boronat P., Cano J.C., Calafate C., Manzoni P. A comparative evaluation of AMQP and MQTT protocols over unstable and mobile networks. Proceedings of the 2015 12th Annual IEEE Consumer Communications and Networking Conference (CCNC).

[68] Alhanahnah M., Yan Q. Towards best secure coding practice for implementing SSL/TLS. Proceedings of the IEEE INFOCOM 2018-IEEE Conference on Computer Communications Workshops (INFOCOM WKSHPS).

[69] Tsao Y.C., Shu C.C., Lan T.S. (2019). Development of a reminiscence therapy system for the elderly using the integration of virtual reality and augmented reality. Sustainability.

[70] Sensors Overview: Android Developers. https://developer.android.com/guide/topics/sensors/sensors_overview.

[71] Roy N., Wang H., Roy Choudhury R. I am a smartphone and i can tell my user’s walking direction. Proceedings of the 12th Annual International Conference on Mobile Systems, Applications, and Services.

[72] Balanis C.A. (1997). Antenna Theory: Analysis and Design.

[73] Jiang J.-R., Subakti H., Chen C.C., Sakai K. PINUS: Indoor Weighted Centroid Localization with Crowdsourced Calibration. Proceedings of the International Conference on Parallel and Distributed Computing: Applications and Technologies.

[74] Jiang J.-R. (2018). An improved cyber-physical systems architecture for Industry 4.0 smart factories. Adv. Mech. Eng..

[75] Mohapatra H. Socio-technical Challenges in the Implementation of Smart City. Proceedings of the IEEE 2021 International Conference on Innovation and Intelligence for Informatics, Computing, and Technologies (3ICT).

[76] Mallinson D.J., Shafi S. (2022). Smart home technology: Challenges and opportunities for collaborative governance and policy research. Rev. Policy Res..

[77] Cavus N., Mrwebi S.E., Ibrahim I., Modupeola T., Reeves A.Y. (2022). Internet of Things and Its Applications to Smart Campus: A Systematic Literature Review. Int. J. Interact. Mob. Technol..

